# Caspase-8-mediated inflammation but not apoptosis drives death of retinal ganglion cells and loss of visual function in glaucomaa

**DOI:** 10.21203/rs.3.rs-4409426/v1

**Published:** 2024-06-18

**Authors:** Yinjie Guo, Bhupender Verma, Maleeka Shrestha, Ann Marshak-Rothstein, Meredith Gregory-Ksander

**Affiliations:** Xiangya Hospital Central South University; Schepens Eye Research Institute of Massachusetts Eye and Ear; Harvard University HSPH: Harvard University T H Chan School of Public Health; University of Massachusetts Chan Medical School Department of Medicine; Schepens Eye Research Institute of Massachusetts Eye and Ear

**Keywords:** Apoptosis, Caspase 8, Fas ligand, Glaucoma, Inflammation, Neuroinflammation, microglia, astrocytes

## Abstract

**Background-:**

Glaucoma is a complex multifactorial disease where apoptosis and inflammation represent two key pathogenic mechanisms. However, the relative contribution of apoptosis versus inflammation in axon degeneration and death of retinal ganglion cells (RGCs) is not well understood. In glaucoma, caspase-8 is linked to RGC apoptosis, as well as glial activation and neuroinflammation. To uncouple these two pathways and determine the extent to which caspase-8-mediated inflammation and/or apoptosis contributes to the death of RGCs, we used the caspase-8 D387A mutant mouse (*Casp8*^*DA/DA*^) in which a point mutation in the auto-cleavage site blocks caspase-8-mediated apoptosis but does not block caspase-8-mediated inflammation.

**Methods-:**

Intracameral injection of magnetic microbeads was used to elevate the intraocular pressure (IOP) in wild-type, Fas deficient Fas^lpr^, and *Casp8*^*DA/DA*^ mice. IOP was monitored by rebound tonometry. Two weeks post microbead injection, retinas were collected for microglia activation analysis. Five weeks post microbead injection, visual acuity and RGC function were assessed by optometer reflex (OMR) and pattern electroretinogram (pERG), respectively. Retina and optic nerves were processed for RGC and axon quantification. Two- and five-weeks post microbead injection, expression of the necrosis marker, RIPK3, was assessed by qPCR.

**Results-:**

Wild-type, Fas^lpr^, and *Casp8*^*DA/DA*^ mice showed similar IOP elevation as compared to saline controls. A significant reduction in both visual acuity and pERG that correlated with a significant loss of RGCs and axons was observed in wild-type but not in Fas^lpr^ mice. The *Casp8*^*DA/DA*^ mice displayed a significant reduction in visual acuity and pERG amplitude and loss of RGCs and axons similar to that in wild-type mice. Immunostaining revealed equal numbers of activated microglia, double positive for P2ry12 and IB4, in the retinas from microbead-injected wild-type and *Casp8*^*DA/DA*^ mutant mice. qPCR analysis revealed no induction of RIPK3 in wild-type or *Casp8*^*DA/DA*^ mice at two- or five-weeks post microbead injection.

**Conclusions-:**

Our results demonstrate that caspase-8-mediated extrinsic apoptosis is not involved in the death of RGCs in the microbead-induced mouse model of glaucoma implicating caspase-8-mediated inflammation, but not apoptosis, as the driving force in glaucoma progression. Taken together, these results identify the caspase-8-mediated inflammatory pathway as a potential target for neuroprotection in glaucoma.

## Background

It is well established in both human and experimental models of glaucoma that apoptosis of retinal ganglion cells (RGCs) is the common endpoint (1–4). However, in the DBA/2J genetic mouse model of glaucoma, specific ablation of the proapoptotic molecule BCL2-associated X protein (BAX) in the RGCs prevented apoptosis of the RGC soma, but the axons continued to degenerate (1), indicating axon degeneration can occur independently of RGC apoptosis. Moreover, there is significant evidence in both human and experimental models of glaucoma demonstrating axon degeneration precedes RGC apoptosis (5–10) and early axon damage in the optic nerve head (ONH) is linked to glial activation and inflammation (11–15).

To further support the idea that inflammation may be a shared pathway that links axon degeneration and death of RGCs, recent studies demonstrate that anti-inflammatory drugs such as minocycline and etanercept prevent both axon degeneration and death of RGCs in experimental models of glaucoma (15–18). However, these drugs are also anti-apoptotic and therefore it is unclear whether blocking inflammation alone can prevent axon degeneration and RGC apoptosis. Along these same lines, our laboratory has demonstrated that blocking the Fas-Fas ligand (FasL) signaling pathway prevents both axon degeneration and death of RGCs in the microbead-induced mouse model of glaucoma, as well as in the spontaneous DBA/2J chronic mouse model of glaucoma (19–21). While triggering of the Fas receptor is best known for inducing apoptosis, Fas activation can also induce the release of proinflammatory cytokines and promote inflammation (22–25). Using a small peptide antagonist of the Fas receptor to block Fas activation in a microbead-induced mouse model of glaucoma, we revealed that blocking Fas activation prevents axon degeneration and death of RGCs, as well as microglia activation and the induction of pro-inflammatory cytokines and chemokines (21). Taken together, these data reveal the essential role the Fas-FasL signaling pathway plays in the pathogenesis of glaucoma and indicate the Fas-FasL signaling pathway mediates both apoptosis of RGCs, as well as glial cell activation and inflammation. However, in these previous studies we could not evaluate the extent to which Fas-mediated apoptosis and/or Fas-mediated inflammation contribute to axon degeneration and death of RGCs since we were unable to uncouple Fas-mediated inflammation from Fas-mediated apoptosis.

While it is known that triggering of the Fas receptor induces apoptosis through the activation of caspase-8, caspase-8 can also induce the production of pro-inflammatory mediators (26–28). Moreover, caspase-8 activation has also been linked to inflammation in experimental models of acute glaucoma and inhibition of caspase-8 blocks inflammation and prevents death of RGCs (29, 30). However, these studies were unable to distinguish the relative importance of caspase 8-mediated apoptosis versus caspase-8-mediated inflammation in axon degeneration and RGC apoptosis.

To better dissect the outcomes of the caspase-8 signaling cascade, the laboratories of David Wallach and Igor Brodsky created two mouse lines in which the auto-cleavage of caspase-8 was abrogated due to a aspartate to alanine substitution at position 387 (31, 32). Using these *Casp8*^*DA/DA*^ mice, they demonstrated the auto-cleavage of activated caspase-8 was required for the induction of apoptosis. However, while abrogating the auto-cleavage of caspase-8 compromised FasL-induced cell death, the mutation had no effect on the non-apoptotic functions of caspase-8 (31, 32). Thus, they were able to effectively uncouple caspase-8-mediated apoptosis from the caspase-8-mediated inflammation.

We have now utilized the *Casp8*^*DA/DA*^ mice to determine the extent to which caspase-8-mediated inflammation and/or capase-8-mediated apoptosis contributes to axon degeneration and the death of RGCs in a microbead-induced mouse model of glaucoma. Comparing wild-type mice to *Casp8*^*DA/DA*^ mutant mice, our results demonstrate that abrogating the caspase-8-mediated apoptosis pathway had no effect on the development of glaucoma, with significant axon degeneration and death of RGCs in both wild-type and *Casp8*^*DA/DA*^ mutant mice. Our results demonstrate that the caspase-8-mediated extrinsic pathway of apoptosis is not required for axon degeneration or the death of RGCs in a microbead-induced mouse model of glaucoma, indicating that caspase 8-mediated inflammation, but not caspase-8-mediated apoptosis is the driving force in the development of glaucoma.

## Methods

### Animals

All animal experiments were approved by the Institutional Animal Care and Use Committee at Schepens Eye Research Institute and were performed under the guidelines of the Association of Research in Vision and Ophthalmology (Rockville, MD). The 8–12-week-old C57BL/6J wild-type mice (Stock number: 00066) and B6.MRL-Faslpr/J Fas receptor-deficient mice (Fas^lpr^, Stock number: 000482) were purchased from Jackson Laboratories (Bar Harbor, ME). The *Casp8*^*DA/DA*^ mouse line was produced by Dr. Igor Brodsky at the University of Pennsylvania Perelman School of Medicine, Philadelphia, Pennsylvania (32). Both *Casp8*^*DA/DA*^ and Fas^lpr^ mice were fully backcrossed to C57BL/6J. The mice were housed and maintained under cyclic light (12L-30 lux:12D) conditions in an AAALAC-approved animal facility at the Schepens Eye Research Institute.

### Genotyping

**Figure 1:**
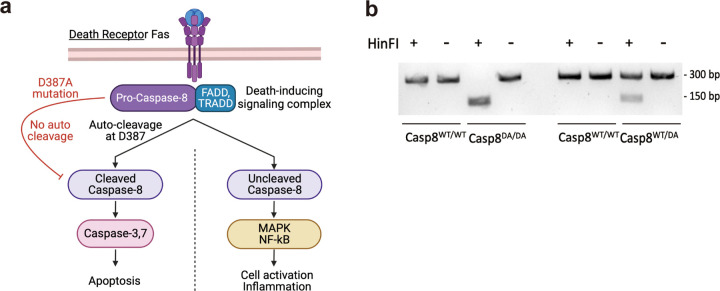
Death receptor signaling via caspase-8 pathway **(a)** A schematic diagram of the Fas-mediated death receptor signaling pathway in wild-type and caspase-8 mutant (*Casp8*^*DA/DA*^) mice. When Fas ligand engages the Fas receptor, pro-caspase-8 is directly recruited to the death-induced signaling complex (DISC) by the adapter proteins FADD and TRADD. Pro-caspase-8 undergoes autocleavage at D387 to produce catalytically active caspase-8 that leads to apoptosis via downstream activation of effector caspases, caspase-3 and caspase-7. The recruitment of pro-caspase-8 to the DISC also upregulates immune cell activation inflammation via MAP-kinase and NF- κB pathways, and autocleavage of caspase-8 is not required for this function. A point mutation at the auto-cleavage site, D387A, prevents caspase-8 activation and blocks caspase-8-mediated apoptosis; however, it does not block caspase-8-mediated inflammation. Schematic created with BioRender.com **(b)** A DNA gel depicting typical genotyping results of a restriction fragment length polymorphism assay showing expected sizes of HinFI undigested (–) and digested (+) PCR amplicon products produced from genomic DNA of mice from the indicated genotypes. Both wild-type and *Casp8*^*DA/DA*^ produce a 300-base pair PCR amplicon and following restriction digestion with HinFI, two fragments of 150-base pair appear in Casp8^DA/DA^ but not in wild-type mice.

The genotype of *Casp8*^*DA/DA*^ mice was confirmed by restriction fragment length polymorphism. Briefly, genomic DNA was isolated from ear punch biopsies and a small fragment of the caspase-8 gene flanking the *Casp8*^*DA/DA*^ mutation was amplified from the genomic DNA using forward primer 5’-GGCCTCCATCTATGACCTGA-3’ and reverse primer 5’- CCAGGAGGCCAAACTTACTG − 3’ that produced a 300 base pairs amplicon from both caspase-8 wild-type and mutant mice. The amplicons were restriction digested using HinFI enzyme (New England Biolabs, Cat# R0155S). The wild-type caspase-8 allele did not have a HinFI site, while the *Casp8*^*DA/DA*^ was digested by HinFI to produce two digested fragments of 150 base pairs (Fig. 1b). The genotyping for Fas^lpr^ was performed by TransnetYX (TransnetYX, Cordova, TN) from ear punch biopsies.

### Magnetic microbead-induced model of elevated intraocular pressure (IOP)

Mice were anesthetized by intraperitoneal injection of a mixture of ketamine (100mg/kg; Ketaset; Fort Dodge Animal Health, Fort Dodge, IA) and xylazine (9 mg/kg; TranquiVed Vedco, Inc., St. Joseph, MO) supplemented by topical application of proparacaine (0.5%; Bausch & Lomb, Tampa, FL) to the eye. IOP elevation was induced by injecting magnetic microbeads (Dynabeads^™^ M-450 Epoxy; Life Technologies, Waltham, MA) into the anterior chamber of the right eye of each animal under a surgical microscope, as previously reported with slight modifications (33). Briefly, magnetic microbeads were processed to remove epoxy residues and resuspended at a concentration of 2×10^6^ beads/μL. The right cornea was gently punctured near the limbus using a sharp glass micropipette (Clunbury Scientific LLC, Bloomfield Hills, MI) mounted on a microinjection system, including a manual micromanipulator and a manual microsyringe pump with a digital display (World Precision Instruments Inc., Sarasota, FL). Magnetic microbeads (2.5 μL) were injected into the anterior chamber using the glass micropipette and were spread to the iridocorneal angle with a magnet (Geomag, 0.45 Tesla magnetic strength). Mice that developed signs of inflammation (clouding of the cornea, edematous cornea), blockage of visual pathway (cataract, microbeads adhered to the endothelium of the central cornea), and IOP not elevated above 18 mmHg at day 7 post injection were excluded from the study.

### IOP measurements

IOP was measured with a rebound Tonolab tonometer (Colonial Medical Supply, Espoo, Finland). Briefly, mice were anesthetized by 3% isoflurane in 100% oxygen delivered with a precision vaporizer. IOP measurement was initiated within 2–3 minutes after the animal lost toe-pinch reflex. Anesthetized mice were placed on a platform, and the tip of the pressure sensor was placed approximately 2 mm from the central cornea. Six IOP measurements were taken, and the average IOP was displayed automatically on the tonometer; this machine-generated mean was considered as one reading, and six such readings were obtained for each eye. All IOPs were taken at the same time of day (between 9:00 am and 12:00 pm) to minimize the IOP variation. Cumulative IOP was calculated using an in-house MATLAB program (MathWorks Inc., Natick MA).

### Optomotor response (OMR)

The visual acuity of mice was measured using an OMR-based spatial frequency threshold test with the Optodrum (Striatech). OMR was performed in awake and unrestrained mice. Mice were placed on a 2-inch-wide circular pedestal located in the center of a testing arena formed by four computer monitors arranged in a square (Fig. 3a). The monitors displayed a moving vertical black and white grating pattern, thus producing a virtual rotating cylinder where rotation speed (12 deg/sec) and contrast (100%) were kept constant throughout the experiment. Right eye was tested on the counterclockwise rotation of the grating and the cycle per degree was adjusted using a preprogrammed staircase method. The software used captured the outline of the mouse, while nose and tail pointers were utilized to automatically evaluate their tracking behavior. Tracking behavior was only recorded when the mice were stationery and tracking was considered positive when there was a reproducible smooth pursuit of the head or rotation of the body in the direction concordant with the stimulus. Trials of spatial frequency were repeated until the presence or absence of the tracking response could be established unequivocally. Confirmation required two positive trials and three negative trials at the next higher (more difficult) cycle per degree. While the software generated the results automatically, individuals blinded to the experimental groups confirmed all tracking behavior and performed all post study measurements.

### Pattern electroretinogram (pERG)

pERG was performed under dim red light. Mice were anesthetized by intraperitoneal injection of a mixture of ketamine (100mg/kg; Ketaset; Fort Dodge Animal Health, Fort Dodge, IA) and xylazine (9 mg/kg; TranquiVed Vedco, Inc., St. Joseph, MO). The mice were placed on a built-in warming plate (Celeris small rodent ERG system, Diagnosys, LLC,) that maintained their body temperature at 37°C in a stable position. One drop of 1% tropicamide (Bausch Health LLC, Bridgewater, NJ, USA) followed by GenTeal (Alcon Laboratories Inc., Fort Worth, Texas, USA) was applied to both corneas, and the light-guided pattern stimulator was placed on the right cornea while a flash stimulator was placed on the left cornea. The pattern displayed on the pattern stimulator consisted of a black and white checkerboard with a check size of 1° at 98% contrast, an intensity of 50 cd.s/m^2^, and a spatial frequency of 0.05 cycles/degree. The data were acquired at a frequency of 1000 Hz, from 50 ms pre trigger to 420 ms post trigger. A total of 300 complete contrast reversals of pERG were repeated twice and 600 cycles were segmented, averaged, and recorded. The averaged pERGs were analyzed to evaluate the P1-N2 amplitude.

### Immunohistochemistry of the whole retinal flat mount

Immediately following euthanasia, eyes were enucleated and fixed in 4% paraformaldehyde for 2 hours at 4 °C. The retinas were dissected out from the eyecup, and four petals were created to produce retinal flat mounts. The retinas were incubated in blocking solution (10% donkey serum, 1% bovine serum albumin, 0.5% Triton X-100 in 1x phosphate buffered saline) for 1 hour at room temperature followed by incubation with primary antibodies against Brn3a, an RGC-specific marker (Millipore Cat# MAB1585, Billerica, MA), and P2ry12, a microglia-specific marker (AnaSpec Inc, Cat# AS-55043A, Fremont, CA) at 4°C for 72 hours. The retinas were then incubated with Alexa Fluor 594 conjugated anti-mouse (Fisher Scientific, Cat# NC0322938) and Alexa Fluor 647 conjugated anti-rabbit (Thermo Fischer Scientific, Cat# A-21245) secondary antibodies at 4°C for 48 hours. Isolectin B4-cojugated Alexa flour 488 (Invitrogen, Waltham, MA, Cat # I21411) was used as a marker for activated microglia and macrophages. Retinas were counter stained with 4′,6-diamidino-2-phenylindole (DAPI), and retinal flat mounts were prepared in permafluor mountant (Epredia, Cat# TA-030-AM) on glass slides.

### Imaging and quantification of retinal ganglion cells

To quantitate retinal ganglion cells, eight non-overlapping images from four petals with two images from each petal were taken using a 40x objective lens on a Leica TCS SP8 confocal microscope system. All Brn3a-stained RGCs were quantitated using an in-house MATLAB program with manual Confirmation. The average number of RGCs in the eight images was used to calculate the RGC density that was represented as cells per square millimeter of the retina. Individuals blinded to the experimental groups performed all RGC counts.

### Quantification of optic nerve axons

To quantify axons, optic nerves were dissected and fixed in Karnovsky’s fixative (2% paraformaldehyde and 2.5% glutaraldehyde in 0.1M sodium phosphate buffer, pH 7.4) overnight. Cross-sections of the optic nerve (1 μm) were taken at 1.0 mm posterior to the globe and stained with 1% P-phenylenediamine (PPD) for evaluation by brightfield microscopy. Six to eight non-overlapping photomicrographs were taken with 100x oil objective from two sections of the optic nerve from each mouse, and two images were taken with 20x objective lens to calculate the area of optic nerve. Using ImageJ (34), axons from each 100x magnification image were counted using the “threshold” and “analyze particles” tools, and area of the optic nerve was calculated using 20x magnification images. The average axon counts and average axon density per square millimeter of the optic nerve were analyzed. Individuals blinded to the experimental groups performed all axon counts.

### Quantification of activated retinal microglia

To quantitate P2RY12- and isolectin B4-positive microglia, image stacks of the retinal flat mounts were acquired using the 20x objective lens (zoom 1.7, 7–15 μm depth in the ganglion cell layer and outer plexiform layer) on a Leica TCS SP8 confocal microscope system. The retina was divided into four quadrants, and one image was acquired at the mid-peripheral region from each quadrant. The number of activated microglia were counted manually, and the average number of activated microglia in the four images was used to calculate the activated microglia density per square millimeter of the retina and data presented as cells per square millimeter. Individuals blinded to the experimental groups performed all microglia quantification.

### Quantitative RT-PCR

RNA was isolated from the whole neural retina using QIAGEN RNeasy Mini Kit (Cat# 74134), according to the manufacturer’s instructions. RNA quality for each sample was analyzed by Agilent RNA 6000 Nano Kit (Agilent Technologies, Inc., Cat#5067 − 1511, Santa Clara, CA) and Agilent 2100 Bioanalyzer (Agilent Technologies, Inc., Santa Clara, CA) following manufacturer’s instructions before cDNA synthesis. A total of 1000 ng RNA was reverse transcribed using Superscript IV VILO master mix (Cat# 11756050, Thermo Fisher, Burlington, MA) according to the manufacturer’s instructions. cDNA was diluted 1:9 and then used as a template for each amplification reaction. Quantitative RT-PCR reactions were performed in a 10 μL volume using the Applied Biosystems^™^ Power SYBR^™^ Green PCR Master Mix (Cat# A46109; Sigma-Aldrich, Burlington, MA) according to the manufacturer’s protocol. The receptor-interacting protein kinase-3 (RIPK3) transcript was amplified using forward primer 5’-CTTGAGGCAGTAGTTCTTGGTGG-3’ and reverse primer 5’- GAAGACACGGCACTCCTTGGTA-3’. PCR cycles consisted of a denaturation step at 95°C for 2 min, followed by 39 cycles of 95°C for 15 s and 60°C for 15 s. Each sample was subjected to melting curve analysis to confirm amplification specificity. Samples were run in duplicate, and each experiment included no-template control wells. Samples were normalized to two house-keeping genes, β2 microglobulin (primers from Biorad, Biorad unique assay ID qMmuCIP0042770) and β-actin (forward primer 5’- TACAGCTTCACCACC − 3’ and reverse primer 5’- ATGCCACAGGATTTC − 3’), and expressed as the relative expression using the δ-delta Ct method (35). Fold changes were calculated with respect to saline-injected control eyes.

### Statistical analyses

Graph Pad Prism 8 (La Jolla, CA, USA) was used to perform statistical analysis of the data. One-way ANOVA and Dunnett’s multiple comparisons test were used for RGC, axon, microglia, and qPCR analyses. Two-way ANOVA and Dunnett’s multiple comparisons test were used for all IOP comparisons. A *p*-value of less than 0.05 was considered statistically significant.

## Results

### Casp8 ^DA/DA^ mutation does not affect microbead-induced elevation of IOP

**Figure 2:**
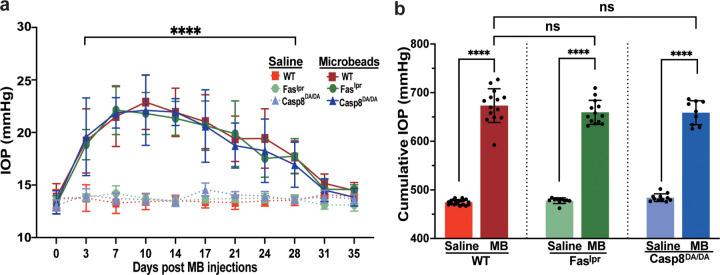
*Casp8*^*DA/DA*^ mutation does not affect magnetic microbead-induced elevated intraocular pressure (IOP) **(a)** A graph showing a 35-day follow-up of mean IOP ± SD (mmHg), and **(b)** cumulative IOP ± SD (mmHg) from saline- or magnetic-microbead-injected WT, Fas-deficient (Fas^lpr^), and *Casp8*^*DA/DA*^ mutant mice.Two-way ANOVA and Dunnett’s multiple comparisons test, **** *p*<0.0001; ns not significant, n=9–14; MB, microbeads; WT, wild-type.

Using a microbead-induced mouse model of glaucoma, we recently demonstrated that axon degeneration and death of RGCs was dependent upon Fas activation and coincided with the induction of caspase-8 and caspase-8-mediated inflammatory cytokines and chemokines (21). Caspase-8 is the initiator caspase downstream of the Fas receptor and can promote both apoptosis and inflammatory gene expression (Fig. 1a) (36). To determine the extent to which caspase-8-mediated apoptosis and/or inflammation contributes to axon degeneration and death of RGCs, we used the *Casp8*^*DA/DA*^ mice in which a mutation in the auto-cleavage site blocks caspase-8-mediated apoptosis, but not caspase-8-mediated inflammation (32). After confirming the genotype of *Casp8*^*DA/DA*^ mice (Fig. 1b), IOP elevation was induced using a magnetic microbead-induced mouse model of glaucoma (33, 37). Wild-type C57BL/6J mice, with intact caspase-8-mediated apoptotic and inflammatory pathways, were used as a positive control, while B6 Fas-deficient (Fas^lpr^) mice, in which FasL cannot induce apoptosis or inflammation, were used as a negative control. A single injection of magnetic microbeads into the anterior chamber resulted in a significant elevation of IOP for up to 35 days in *Casp8*^*DA/DA*^, wild-type, and Fas^lpr^ mutant mice as compared to saline controls (Fig. 2a). There was no significant difference in the time course and magnitude of IOP (Fig. 2a) nor the cumulative IOP (Fig. 2b) among the 3 mouse strains, thereby confirming that Fas-FasL activation of caspase-8-mediated signaling does not affect IOP elevation in the microbead-induced model of glaucoma.

### Loss of visual acuity and RGC function is not dependent on caspase-8-mediated apoptosis

**Figure 3:**
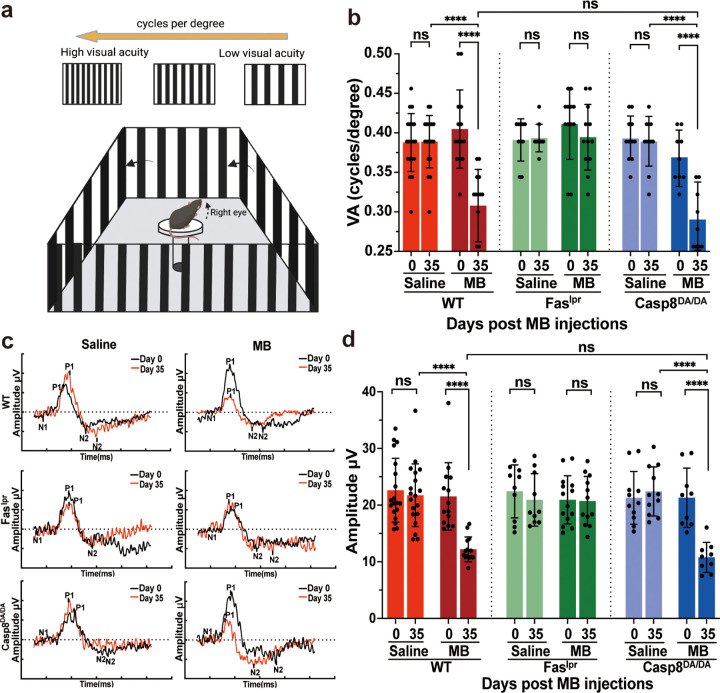
Caspase-8-mediated inflammation, not apoptosis, underlies loss of visual function in glaucoma **(a)** A schematic representation of optomotor response (OMR) apparatus used to measure visual acuity (VA) in cycles/degree. The vertical lines rotated counterclockwise to measure VA in the right eye, while the mouse was placed on a pedestal at the center of OMR apparatus. The OMR measured visually-induced head tracking behavior of mice that occurred when the mouse pursued a sequential rotating black-and-white stripes stimulus pattern shown on four screens. **(b)** A plot showing VA (in cycles/degree) at baseline (day 0) and endpoint (day 35) from saline- or microbead-injected mice from WT, Fas-defcient (Fas^lpr^), and *Casp8*^*DA/DA*^ mutant mice as indicated. **(c)** Graphs showing representative pattern electroretinogram (pERG) amplitude, as a function of time (ms), recordings from saline-(left panels) and microbead-(right panels) injected WT (top panels), Fas^lpr^ (middle panels) and *Casp8*^*DA/DA*^ (bottom panels) mice as labelled. The difference between peak P1 and trough N2 was used to calculate pERG amplitude (μV). **(d)** A plot showing pERG amplitude (μV) at baseline (day 0) and endpoint (day 35) from saline- or microbead-injected mice from WT, Fas-defcient (Fas^lpr^), and *Casp8*^*DA/DA*^ mutant mice as indicated. Data presented as mean ± SD, one-way ANOVA and Dunnett’s multiple comparisons test, **** *p* <0.0001, ns not significant; n=9–16; MB, microbeads; WT, wild-type.

To determine the effects of caspase-8-mediated apoptosis and inflammatory pathways on visual acuity and RGC function, we assessed visual acuity by OMR (Fig. 3a and 3b) and RGC function by pattern electroretinogram (pERG) (Fig. 3c and 3d). At five weeks post microbead injection, a significant reduction in visual acuity was observed in wild-type but not in Fas^lpr^ mice when compared to baseline or saline controls (Fig. 3b). In the absence of Caspase-8-mediated apoptosis, we also found a similar significant reduction in visual acuity in microbead-injected *Casp8*^*DA/DA*^ mice when compared to baseline and saline controls (Fig. 3b). Likewise, at five weeks post microbead injection, a significant reduction in the pERG amplitude was also observed in wild-type but not in Fas^lpr^ mice when compared to baseline or saline controls (Fig. 3c and 3d). Again, microbead-injected *Casp8*^*DA/DA*^ mutant mice presented with a significant reduction in pERG amplitude similar to that observed in wild-type mice, when compared to baseline or saline controls (Fig. 3c, 3d). The equivalent loss of visual acuity and pERG amplitude in both microbead-injected wild-type mice and Casp8^DA/DA^ mice indicate the loss of visual acuity and RGC function in the microbead-induced model of glaucoma is not dependent on caspase-8-mediated apoptosis.

### RGC death and axon degeneration are not dependent on caspase-8-mediated apoptosis

**Figure 4:**
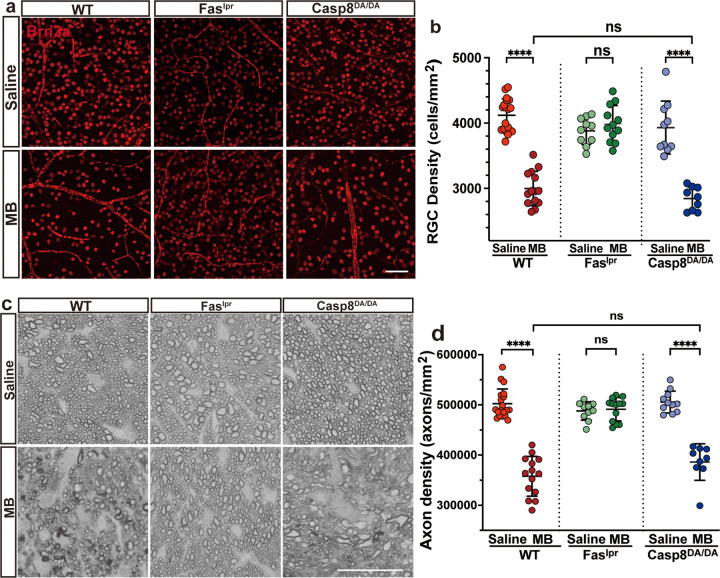
RGC loss and axon degeneration are not dependent on caspase 8-mediated apoptosis Representative **(a)** confocal microscopy images of retinal flat mounts stained for retinal ganglion cells (RGCs) with Brn3a (scale bar 50μm) and **(c)** brightfield images of optic nerve sections stained for axons with P-phenylenediamine (PPD, scale bar 50μm) from saline-(top panels) and microbead-(bottom panels) injected WT, Fas^lpr^, and *Casp8*^*DA/DA*^ mice showing RGC and axon loss in microbead-injected WT and *Casp8*^*DA/DA*^ but not in Fas^lpr^. Graphs showing **(b)** RGC density data presented as RGC number per square mm retina ± SD and **(d)** axon density data presented as axon number per square mm of ON ± SD. One-way ANOVA and Dunnett’s multiple comparisons test, **** *p* <0.0001, ns not significant; n=10–20; MB, microbeads; WT, wild-type.

The magnetic microbead-induced elevation of IOP leads to axon degeneration and death of RGCs as early as 3-weeks post-microbead injection (33). To determine the extent to which caspase-8-mediated apoptosis contributes to axon degeneration and death of RGCs, quantification of RGC and axon density was performed at 5 weeks post microbead injection. RGC density was assessed in retinal whole mounts stained with anti-Brn3a, a RGC-specific antibody (Fig. 4a). As expected, quantification of Brn3a-stained RGCs revealed a significant decrease in RGC density in microbead-injected wild-type but not Fas^lpr^ mice as compared to saline controls (Fig. 4b). Interestingly, a significant decrease in RGC density was also observed in the Casp8^DA/DA^ mutant mice when compared to saline controls (Fig. 4a, 4b). Moreover, there was no significant difference in the magnitude of RGC loss when comparing microbead-injected *Casp8*^*DA/DA*^ mutant mice to microbead-injected wild-type mice. Similar to the RGC analysis, at 5 weeks post microbead injection optic nerve sections stained with PPD also revealed a significant decrease in axon density in wild-type but not in Fas^lpr^ mice when compared to saline controls (Fig. 4c, 4d). Moreover, there was no significant difference in the magnitude of axon loss when comparing microbead-injected *Casp8*^*DA/DA*^ mutant mice to microbead-injected wild-type mice. In addition, there was no significant difference in the optic nerve cross-sectional area between any of the mouse strains following saline or microbead injections (Fig. S1b). Quantification of total axons per optic nerve also revealed a significant loss of axons in microbead-injected wild-type and *Casp8*^*DA/DA*^ mutant mice but not in Fas^lpr^ mice when compared to saline controls (Fig. S1b). Taken together, these results indicate that caspase-8-mediated apoptosis is not required for axon degeneration and death of RGCs in the microbead-induced mouse model of glaucoma.

#### Significant microglia activation in both microbead-injected wild-type and Casp8^DA/DA^ mutant mice

**Figure 5:**
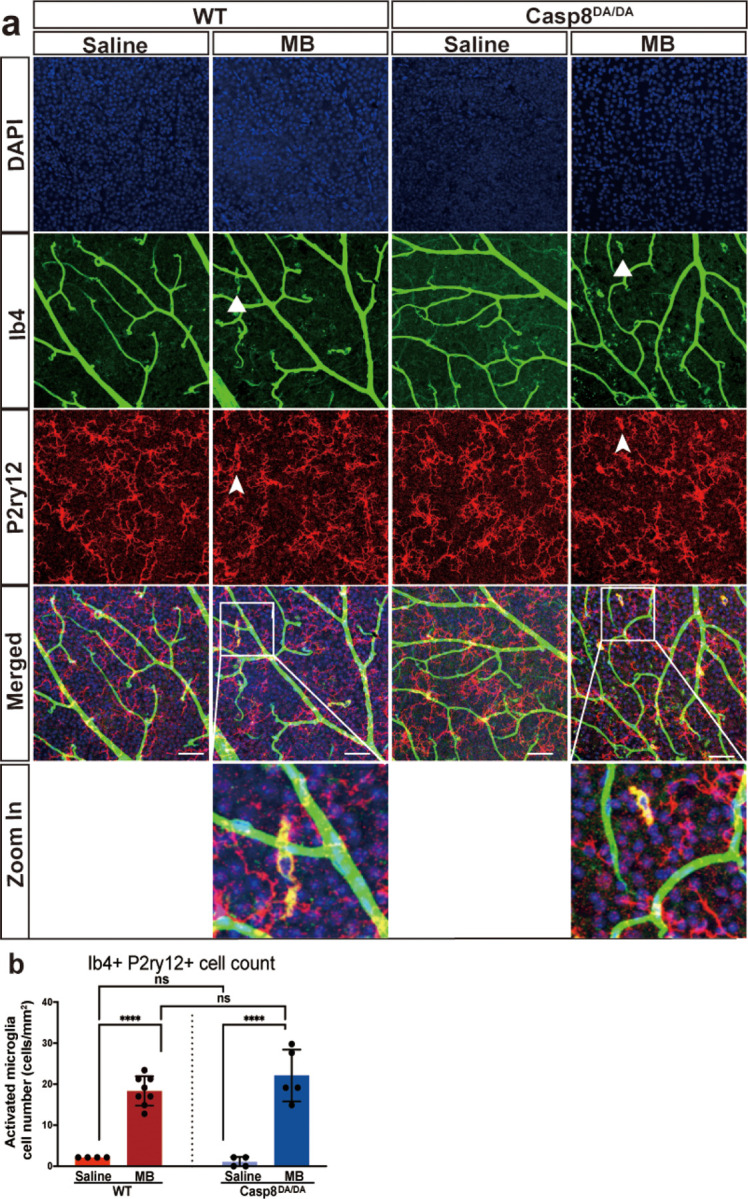
*Casp8*^*DA/DA*^ mutation does not affect microglia activation post-elevated intraocular pressure **(a)** Representative confocal microscopy images of retinal flat mounts co-stained with P2Ry12, isolectin-B4 (IB-4), and DAPI for activated microglia at day 14 post-microbead or saline injection from WT and *Casp8*^*DA/DA*^ mice. **(b)** Graph showing quantification of P2RY12 and IB4 double positive microglia, revealing significant increase in activated microglia in both WT and casp8^DA/DA^ mutant mice as compared to that in saline controls. Data represented as mean activated microglia cell number per square mm of retina ± SD. n=4–5, One-way ANOVA and Dunnett’s multiple comparisons test, ****p <0.0001; ns not significant; MB, microbeads; WT, wild-type; scale bar 50 μm.

It is well established that microglia contribute to glaucoma onset and progression, and the extent of microglia activation correlates with the extent of RGC death and optic nerve degeneration (16, 38). Moreover, caspase-8 signaling has been shown to control microglia activation and neurotoxicity in other models of neurodegeneration (26). To evaluate retinal microglia activation following elevated IOP, retinal whole mounts were prepared from wild-type and *Casp8*^*DA/DA*^ mutant mice at two weeks post microbead injection. The retinal whole mounts were stained with P2ry12, a microglia-specific marker and isolectin-B4 (IB4), a marker for activated microglia (39, 40) and activated microglia stained double-positive with P2ry12 and IB4 (Fig. 5a). Immunostaining revealed activated microglia in the retinas from microbead-injected wild-type and *Casp8*^*DA/DA*^ mutant mice, while no activated microglia were observed in the retinas from the saline-injected controls (Fig. 5a). Quantification of double-positive cells demonstrated a significant increase in the number of activated microglia in the retinas of both *Casp8*^*DA/DA*^ and wild-type mice at 2 weeks post microbead injection (Fig. 5b). Together, these data demonstrate the absence of caspase-8 autocleavage in *Casp8*^*DA/DA*^ mice had no effect on microglia activation following elevated IOP.

### Blocking Caspase-8-mediated apoptosis does not shift cell death to necroptosis

**Figure 6:**
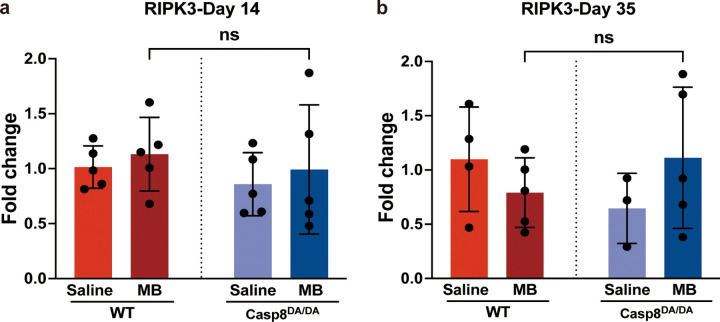
*Casp8*^*DA/DA*^ mutation does not shunt RGC death to RIPK3-mediated necroptosis Graphs showing fold change in RIPK3 transcript levels as assessed by quantitative reverse transcription PCR (RT-qPCR) in WT and Casp8DA/DA mice at **(a)** early, day 14, and **(b)** late, day 35, timepoints following elevation of intraocular pressure. N=4–5, One-way ANOVA and Dunnett’s multiple comparisons test; ns not significant; MB, microbeads; WT, wild-type.

Caspase-8 together with cellular FLICE-like inhibitory protein (cFLIP) prevents receptor-interacting protein kinase-3 (RIPK3) mediated necroptosis (41–43) and knocking out caspase-8 is embryonically lethal (44). Therefore, because no reduction in RGC death was observed following elevated IOP in *Casp8*^*DA/DA*^ mutant mice, we sought to determine if blockage of the caspase-8-mediated apoptotic pathway shifted RGC death from apoptosis to necroptosis. At 2 and 5 weeks post-microbead injection qPCR was performed to assess transcript levels of RIPK3 in neural retina. The qPCR analysis revealed no induction of RIPK3 in wild-type or *Casp8*^*DA/DA*^ mice at 2- or 5-weeks post microbead injection (Fig. 6a and 6b). These results indicate that in the absence of caspase-8-mediated apoptosis, RGC death did not shift from apoptosis to necroptosis in *Casp8*^*DA/DA*^ mutant mice and this is in agreement with previous studies demonstrating that while the non-cleavable caspase-8 in *Casp8*^*DA/DA*^ mutant mice is impaired in inducing apoptosis, it remains capable of blocking necroptosis (31, 41, 45).

## Discussion

The mechanisms underlying axon degeneration and RGC death in glaucoma are complex and multifactorial. It is well established that FasL plays a key role in the pathogenesis of glaucoma and that RGCs die by apoptosis in both human and experimental models of glaucoma (2, 3, 32). Nevertheless, approaches that specifically target the apoptotic pathway in RGCs are only partially protective (1, 46). Together, these data suggest that FasL may function beyond simply inducing RGC apoptosis. Apart from its well-documented capacity to trigger cell death, FasL is also a potent inducer of proinflammatory cytokine production (22, 47). Caspase-8, a critical component of the Fas signaling cascade, has been implicated in the pathogenesis of glaucoma as a mediator of both FasL-induced apoptosis and inflammation (48), two distinct processes that potentially promote the demise of RGCs. To better understand the mechanism responsible for RGC death, we have now utilized a gene-targeted mouse line with an inactivating point mutation in the auto-cleavage site of caspase 8, D387A. This *Casp8*^*DA/DA*^ mutation dramatically reduces caspase-8-mediated apoptosis but does not interfere with caspase-8-mediated inflammation (32). By combining the Casp8^DA/DA^ mutant mice with the microbead-induced mouse model of glaucoma, we now find that RGCs do not die by caspase-8-mediated extrinsic apoptosis. Caspase-8-mediated inflammation, not caspase-8-mediated apoptosis, drives axon degeneration and RGC death in experimental glaucoma.

The two major apoptotic pathways, intrinsic or mitochondrial-mediated and extrinsic or death receptor-mediated have both been implicated in human and experimental models of glaucoma (1, 49–53). The intrinsic pathway of apoptosis is a mitochondria-mediated pathway where activation of pro-apoptotic proteins such as Bax and Bak initiates mitochondrial changes and activation of the initiator caspase, caspase-9 (54). The main evidence that RGCs die via the intrinsic apoptotic program stems from studies using Bax deficient mice where death of RGCs is completely eliminated in the DBA/2J mouse model of glaucoma, as well as other glaucoma-relevant models, including optic nerve crush and axotomy (1, 55, 56). However, subsequent studies also implicate the death-receptor mediated extrinsic apoptotic pathway, where inhibition of the initiator caspase, caspase-8, protects RGCs in a mouse model of acute glaucoma (29, 30), as well as the microbead-induced model of glaucoma (48). However, the caspase-8 inhibitor used in these studies, Z-IETD-FMK, while a potent inhibitor of caspase-8, also shows cross reactivity with caspase-3 and caspase-9 (57, 58), making it unclear if the caspase-8-mediated extrinsic pathway of apoptosis is actually involved in the death of RGCs in glaucoma. In our current study using the Casp8^DA/DA^ gene-targeted mouse line where caspase-8-mediated apoptosis is specifically impaired, we found no reduction in the death of RGCs in microbead-injected Casp8^DA/DA^ mutant mice when compared to microbead-injected WT mice. Therefore, we conclude the caspase-8-mediated extrinsic apoptotic pathway is not involved in the death of RGCs in the microbead-induced mouse model of glaucoma.

A significant complication arising from knocking out caspase-8 or inhibiting caspase-8 activity is the unintended induction of necroptosis (41, 42, 48). In addition to the induction of apoptosis, caspase-8 also forms a catalytically active complex with cFLIP that prevents RIPK3 dependent necroptosis, and knocking out or inhibiting caspase-8 activity can lead to necrotic cell death (41, 42, 48). However, previous studies demonstrated that while the non-cleavable caspase-8 in Casp8^DA/DA^ mutant mice is impaired in inducing apoptosis, it remains capable of blocking necroptosis (31, 41, 45). In our current study, despite the absence of caspase-8-mediated apoptosis in the Casp8^DA/DA^ mutant mice, qPCR showed no induction of RIPK3 at day 14 or Day 35 post microbead injection in Casp8^DA/DA^ mutant mice confirming that RGC death was not being shunted from apoptosis to necroptosis.

In addition to acting as a protease that promotes effector caspase activation resulting in apoptosis, caspase-8 can also serve as a scaffold for assembly of an NF*κ*B -activating complex leading to inflammation and several recent studies link caspase-8 activation with neurotoxic inflammation in glaucoma (29, 30, 48, 59). However, inhibitors of caspase-8 activation block both the apoptotic and inflammatory functions of caspase-8 making it impossible to specifically study the role of caspase-8-mediated inflammation in the pathogenesis of glaucoma. Using the Casp8^DA/DA^ gene-targeted mouse line we were able to successfully uncouple the two pathways, where the mutation in the autocleavage site only compromises caspase-8-mediated apoptosis but has no effect on the non-apoptotic functions of caspase-8 (31, 32). The complete absence of neuroprotection in the microbead-injected Casp8^DA/DA^ mutant mice implicates the caspase-8-mediated inflammatory pathway, not the caspase-8-mediated apoptotic pathway as a key signaling pathway in the pathogenesis of glaucoma.

The results of our current study combined with previous work by Libby et al., (1) support the conclusion that in glaucoma, RGC apoptosis is dependent upon Bax and mediated through the intrinsic apoptotic pathway. However, accumulating evidence demonstrates axon damage precedes apoptosis of the RGC soma (5–10) and this axon damage is linked to glial activation and the production of inflammatory and neurotoxic mediators that can directly damage the axons (11–16). In human and experimental models of glaucoma, activated astrocytes (60–63) and activated microglia (11–16) are detected in the optic nerve head (ONH) and the extent of microglia activation in the ONH coincides with the severity of axon degeneration in the DBA/2J mouse model of glaucoma (15). We demonstrated previously in an inducible mouse model of glaucoma that microglia activation and the induction of caspase-8 and caspase-8 mediated inflammatory cytokines and chemokines, as well as axon degeneration and death of RGCs is dependent upon Fas activation (21). However, astrocytes have also been identified as potential drivers of RGC death in mouse models of optic nerve crush and glaucoma (64, 65) with the caspase-8 signaling pathway identified as the mediator of astrocyte neurotoxicity in a microbead-induced rat model of glaucoma (48). Moreover, astrocytes have also been shown to be resistant to FasL-induced apoptosis and instead become reactive and produce proinflammatory cytokines (25, 66). Together, these studies highlight the importance of glial activation and inflammation in axon degeneration and reveal a link between Fas activation, caspase-8, and glial neurotoxicity in glaucoma. Future work will focus on elucidating the mechanism(s) by which Fas-mediated activation of caspase-8 controls astrocyte and microglia neurotoxicity in glaucoma.

## Conclusions

Our results demonstrate that caspase-8-mediated apoptosis is not required for axon degeneration or death of RGCs in a microbead-induced mouse model of glaucoma, indicating that caspase 8-mediated inflammation, but not caspase 8-mediated apoptosis is the driving force in glaucoma progression. Taken together, these results identify the caspase-8-mediated inflammatory pathway as potential target for neuroprotection in glaucoma.

## Data Availability

The datasets generated and/or analyzed during the current study are available from the corresponding author on reasonable request.

## Abbreviations

AAV2: adeno-associated virus serotype 2
BAX: BCL2 associated X
cFLIP: cellular FLICE-like inhibitory protein
CRISPR: clustered regularly interspaced short palindromic repeats
DAPI: 4′,6-diamidino-2-phenylindole
DISC: death-inducting signaling complex
IB4: isolectin-B4
IL-1β: interleukin-1 β
IOP: intraocular pressure
MIP: Lens Fiber Major Intrinsic Protein
NF-κB: nuclear factor- kappa B
OMR: optomotor response
ONH: optic nerve head
pERG: pattern electroretinogram
PPD: P-phenylenediamine
RGCs: retinal ganglion cells
RIPK3: receptor-interacting protein kinase-3
RT-qPCR: quantitative reverse transcription PCR
TNFR: tumor necrosis factor receptor
TRAIL: TNF-Related Apoptosis Inducing Ligand
